# Application of behavioral economics principles to reduce injectable contraceptive discontinuation in rural Ethiopia: A stratified-pair, cluster-randomized field trial

**DOI:** 10.12688/gatesopenres.12987.2

**Published:** 2019-11-20

**Authors:** Ali Mehryar Karim, Dana Guichon, Bantalem Yeshanew Yihun, Nebreed Fesseha Zemichael, Karina Lorenzana, Jeremy Barofsky, Wuleta Betemariam

**Affiliations:** 1 Global Development, Bill & Melinda Gates Foundation, Addis Ababa, Ethiopia; 2 ideas42, New York, New York, 10004, USA; 3 The Last Ten Kilometers Project 2020, JSI Research & Training Institute, Inc., Addis Ababa, Ethiopia; 4 ideas42, Washington, DC, 20005, USA

**Keywords:** Behavioral economics intervention, injectable contraceptive discontinuation, family planning

## Abstract

**Background:** Contraceptive prevalence in Ethiopia jumped from 6% in 2000 to 36% in 2016, mainly due to increased injectable method use. However, discontinuation rates among injectable users were high (38%). Given that the public sector is the major source for injectable contraceptives, John Snow Inc. (JSI) in collaboration with ideas42 worked with Ethiopia’s flagship Health Extension Program to apply behavioral design to mitigate discontinuation of injectable contraceptives.

**Methods:** We applied behavioral economics insights to mitigate the discontinuation of injectable contraceptives. This process created an intervention package, consisting of a health worker planning calendar, a client counseling job aid, and client appointment cards. A stratified-pair cluster-randomized field trial tested the effectiveness of the intervention. The study area included two districts from the four regions where JSI was implementing a family planning program. One district from each region was randomly allocated to the intervention arm. Women visiting health posts to use injectable contraceptives were enrolled in the study. Regression methods, adjusted for study design, participants’ backgrounds, and contextual factors, estimated the intervention’s effect on discontinuation rates.

**Results:** A behavioral design methodology was feasibly implemented in a rural, low-resource setting in Ethiopia. The resultant intervention package was successfully delivered in 19 satellite health posts in four districts. Intervention adherence was high for the appointment cards and counseling job aid, but not for the planning calendar. The injectable discontinuation rate was 10.8 % (95% confidence interval: 2.2, 19.3) points lower in the intervention area compared to the control area during the post-intervention follow-up survey.

**Conclusion:** The use of two tools informed by behavioral economics —the appointment card and counseling job aid—effectively decreased injectable discontinuation even with the presence of other health system bottlenecks. Behavioral economics insights and the behavioral design methodology have the potential to enhance family planning programs in Ethiopia and elsewhere.

## Abbreviations

BE                     Behavioral Economics

CPR                  Contraceptive Prevalence Rate

CBDDM           Community-Based Data for Decision Making

DHS                 Demographic and Health Survey

FMOH             Federal Ministry of Health of the Government of Ethiopia 

FP                    Family Planning

HEW               Health Extension Worker

HEP                 Health Extension Program

IUCD               Intra Uterine Cervical Device

JSI                   John Snow, Incorporated

L10K               Last Ten Kilometers

LAFP               Long Acting Family Planning

MNCH             Maternal, Newborn and Child Health

PHCU              Primary Health Care Unit

RMNCH          Reproductive, Maternal, Newborn and Child Health

SNNP              Southern Nations, Nationalities and Peoples’

WDA              Women Development Army

## Introduction

The national family planning program in Ethiopia has made considerable strides in providing access to services across the country. The contraceptive prevalence rate (CPR) in Ethiopia jumped from 6% in 2000 to 36% in 2016. This increase was mainly the result of a sharp increase in the use of injectable contraceptive methods, from 3% in 2000 to 23% in 2016. Nonetheless, 35% of contraceptive users discontinued use within 12 months of uptake, while the discontinuation rate among injectable contraceptive users was 38% ( Central Statistical Agency and ICF, 2016).

The Ethiopian Federal Ministry of Health (FMOH) aims to increase the CPR to 55% by 2020 ( Federal Ministry of Health, 2015). High rates of discontinuation pose a threat to the achievement of this target, as well as to the intentions of many Ethiopian women to space births and to limit the number of children they have. High rates of contraceptive discontinuation impose a cost on the woman, her family, and her country’s health system. Discontinuation can lead to unintended pregnancies, causing social, economic, and emotional distress, and also contribute to maternal morbidity and mortality ( Ahmed *et al*., 2012; Ali *et al*., 2012; Cleland *et al*., 2012).

Women may choose to discontinue a contraceptive method because their reproductive intentions have changed, and they no longer intend to use family planning. However, if women are discontinuing, but still have intentions to use contraception, the system has not met their needs. Discontinuation rates may be high for a variety of reasons. Some of these are problems at the system level—for example, high rates of stockouts or distance from the source of the family planning method could be structural barriers impeding continued use. However, various nonstructural factors may also contribute. Often, the dominant reason for stopping a modern, reversible method is dissatisfaction ( World Health Organization, 2012). Dissatisfaction with the method could stem from gaps in counseling that lead to suboptimal initial method choices, health concerns or side effects, or choice of an inconvenient method, among other reasons ( Bradley *et al*., 2009). In contexts where structural barriers have for the most part been eliminated, nonstructural, or behavioral, factors may be driving discontinuation. Ethiopia is likely to be such a context. The introduction of the country’s flagship Health Extension Program (HEP) has brought family planning services to the doorsteps of the rural population ( Admasu *et al*., 2016).

Behavioral economics (BE) offers a promising toolkit for designing interventions to change health-related behaviors and decision-making, from both the provider and the client perspective, by improving understanding of why people behave as they do and what motivates their decision-making and action ( Buttenheim & Asch, 2013; Datta & Mullainathan, 2014). Contraceptive discontinuation has been identified as a critical reproductive health challenge that is persistent across contexts and may be responsive to behavioral economics interventions. Contraceptive discontinuation is especially relevant for a behavioral economics lens because there is an explicit gap between stated preferences and behavior ( Ashton *et al*., 2015).

Compounding the cognitive factors surrounding contraceptive use, recent behavioral science research suggests that individuals living in poverty experience a chronic state of scarcity, which imposes additional cognitive demands that make forward-looking decisions and actions difficult ( Mani *et al*., 2013; Shah *et al*., 2012). Individuals experiencing scarcity tend to “tunnel”; focusing on what is most urgent or most salient, displacing all else. This may have harmful effects on future-looking decisions or actions like those involved in contraceptive use. In low-income countries, health workers as well may experience diverse forms of scarcity, living in poverty themselves and working in contexts characterized by large catchment areas, heavy workloads, personnel shortages, and limited time or task management resources.

Behavioral economics offers many promising solutions to address these types of behavioral challenges. Reminders have proven effective in promoting medication adherence and medical appointment attendance across several contexts ( Kannisto *et al*., 2014; Lindenmeyer *et al.*, 2006). Job aids are another widely used behavioral solution that has been effective in improving family planning counseling ( Kim *et al.*, 2007; Leon *et al.*, 2005). Insights from BE and a structured behavioral design methodology have recently been used to improve reproductive health programming ( Buttenheim *et al.*, 2019; McConnell *et al.*, 2018; Spring *et al.*, 2016), suggesting that the approach could be applied to mitigate the contraceptive discontinuation problem in Ethiopia. Further application is needed to understand how insights from behavioral economics can be applied to solve persistent public health challenges particularly in low-resource settings where scarcity increases the likelihood of intention-action gaps. This paper contributes to this understanding by sharing the experience and results of the application of behavioral economics to a family planning program in rural Ethiopia.

Abandonment of injectable methods is an urgent matter, given that they are the primary method of choice among married women of reproductive age in Ethiopia, and that the government sector provides 82% of contraceptive methods ( Central Statistical Agency and ICF, 2016). The Last Ten Kilometers (L10K) Project of John Snow Research & Training Institute, Inc. (JSI) collaborated with ideas42 to work with the HEP to design and test behavioral approaches to mitigate the problem of discontinuation of injectable contraceptives.

The overall goal of this study is to increase the rate of contraceptive continuation among women of reproductive age who are new users of injectable contraceptives in rural Ethiopia. The primary objectives in support of this goal are 1) to use behavioral insights to develop a package of interventions that will improve the rate of contraceptive continuation of women using injectable contraceptives in Ethiopia and 2) to trial this package of interventions and assess the preliminary impact of this package and its limitations.

## Methods

### Study context

The health care delivery system of rural districts in Ethiopia is comprised of three to four primary health care units (PHCUs) supported by one primary hospital. A PHCU is formed by one health center for every 25,000 people in the district, with five satellite health posts ( Federal Ministry of Health, 2015). Health centers are staffed by health officers, nurses, midwives, and laboratory technicians, each with an undergraduate diploma or health degree. They provide preventive and curative services, including basic emergency obstetric and newborn care, as well as supervision of satellite health posts.

The health posts employ two female Health Extension Workers (HEWs) serving a community (kebele) of about 5,000 people with basic community-based promotive, preventive, and curative health services. This system forms the nucleus of the flagship HEP in Ethiopia ( Wakabi, 2008). A network of Women’s Development Army (WDA) volunteers supports the HEWs to ensure community engagement in the delivery of HEP services.

The WDA network is organized into groups of 30 households led by one WDA team leader, with subgroups of six households led by one WDA member ( Admasu *et al*., 2016).

The health posts provide condoms, oral contraceptive pills, emergency contraception, injectable contraceptives, and Implanon insertion services; health centers offer intrauterine contraceptive devices (IUCDs) and implant insertion and removal services in addition to the methods provided at health posts. Permanent methods are offered at primary hospitals or higher-level facilities.

The L10K program sought innovative, community-based strategies for the HEP to provide high-impact reproductive, maternal, newborn, and child health (RMNCH) services. To this end, L10K partnered with 10 local civil society organizations to enhance the interactions between HEP frontline health workers (HEWs and WDAs) and communities to achieve more accessible, efficient, and equitable maternal, newborn, and child health (MNCH) services ( Darmstadt *et al*., 2013). The L10K intervention area covered 115 districts in the Amhara, Oromia, Southern Nations, Nationalities and Peoples’ (SNNP), and Tigray regions, reaching a population of 17 million people, approximately 19% of Ethiopia’s population.

In eight of its 115 intervention districts (two districts per region), L10K collaborated with the respective Regional Health Bureaus to implement interventions to improve the demand for and quality of community-based long-acting family planning (LAFP) services. The eight districts were purposely selected by the respective Regional Health Bureaus to avoid duplication of other partners' work. The family planning interventions began in 2014 and were implemented in one of the four PHCUs within each of the eight selected districts. Intervention activities included family planning counseling training for HEWs, comprehensive family planning for the health center service providers, supplies of national tools and job aids for HEWs, institution of quarterly review meetings, and enhancement of referral and linkages with the health center and health posts for LAFP and postpartum family planning services. The eight PHCUs where L10K was implementing the FP interventions were selected for participation in the BE study.

### The intervention

**Figure 1.**
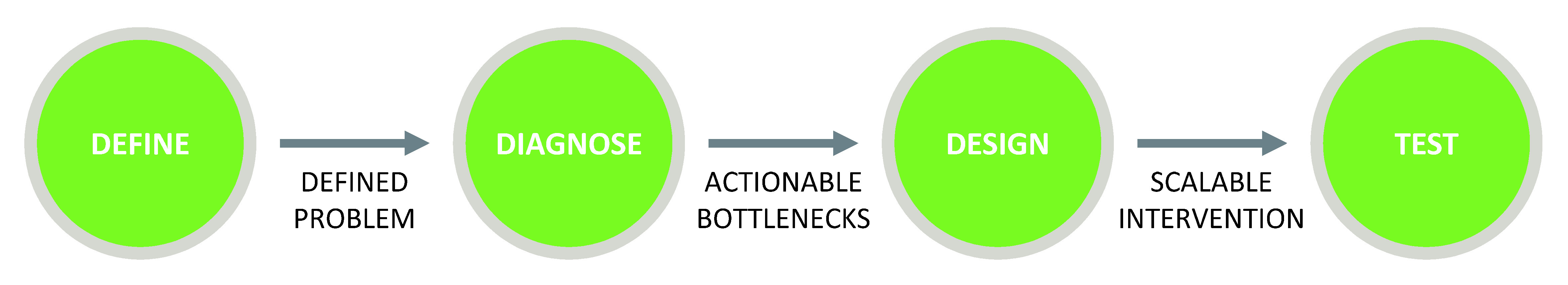
Stages of Behavioral Design methodology ( Datta & Mullainathan, 2014).

To identify behavioral barriers impeding continued use of injectable contraceptives, design a targeted solution, and test the impact of this approach, the study team applied a four-stage behavioral diagnosis and design methodology. This process involved four distinct phases depicted in Figure 1: problem definition, behavioral diagnosis, intervention design, and testing ( Datta & Mullainathan, 2014).

***Problem definition (Step 1).*** The general problem of contraceptive discontinuation is broad, and it encapsulates diverse methods, subpopulations, and manifestations of discontinuation. Thus, the goal of the problem definition process was to (a) identify the different problem definition candidates that represent these multiple dimensions and components of discontinuation in rural Ethiopia, and (b) evaluate these candidate definitions systematically along with a set of criteria so that the strongest and most actionable problem or problems are identified. The criteria were:

1. The key drivers for discontinuation of contraceptives included behavioral issues among others (e.g., cultural issues, problems with the supply chain).
2. The existence of an intention-action gap, i.e., women have the desire to continue using contraceptives, but fail to follow through on this intention and discontinue.
3. The problem could be moved by a behavioral intervention; and,
4. Structures were in place for implementing cluster-randomized clinical trials to test the effectiveness of the behavioral intervention.

Problem definition included the analysis of 2011 Ethiopian Demographic and Health Survey, informal conversations with L10K senior and mid-level managers, process mapping, preliminary behavioral mapping, and a field visit in October 2014. The field visit included conversations with frontline HEP workers (i.e., HEWs and the WDA) and contraceptive users, along with observation of family planning provision.

The process gathered anecdotal evidence that both passive (unconscious decisions) and active (conscious decisions) contraceptive discontinuation could be occurring. All long-acting (i.e., IUCD and implants) and short-acting (i.e., injectables, pills, and condoms) reversible methods may be subject to active discontinuation, while only short-acting reversible contraceptive methods may be subject to passive discontinuation. Several candidates for defining the problem were identified and evaluated based on the four criteria described above. The final problem definition from the process was: “Women of reproductive age who are using injectable contraceptives discontinue use within 12 months of uptake.”

***Behavioral diagnosis (Step 2).*** The objective of the behavioral diagnosis was to identify the primary behavioral drivers or the “behavioral bottlenecks” leading to discontinuation of injectable contraceptives within the study population. The process included detailed behavioral mapping to generate hypothesized bottlenecks; contextual reconnaissance to gather evidence; and synthesis of evidence and evaluation of hypothesized bottlenecks. Evidence gathered during the contextual reconnaissance stage included data from client and provider interviews, observations of family planning provision, analysis of data from Demographic and Health Surveys, conversations with project staff, and analysis of secondary literature. This evidence was used to confirm, reject, or refine the hypothesized bottlenecks.

The set of hypotheses that described the primary bottlenecks driving observed behavior (i.e.: discontinuation of injectable contraceptives) and its intervention design implications are presented in three categories: bottlenecks contributing to passive discontinuation, active discontinuation, and provider behavior ( Table 1).

**Table 1.** Overview of behavioral bottlenecks and their design implications.

Behavioral Bottlenecks	Design Implications
***Bottlenecks Contributing to Passive Discontinuation***	
1. Prospective Memory Failure to Follow Through on Next Injection: Due to prospective memory failure, women do not come to the health post at the appropriate time.	Salient, timely reminders for women.
2. Tunneling Leads to Myopic Planning or Failures to Plan for Next Injection: Women tunnel on the specific date of a follow-up appointment, yet they do not form any contingency plans for receiving their injection if they cannot make that exact date.	Prompt plan-making and contingency planning for return appointments.
3. Hassle Factors: Women may be deterred by hassles associated with successful continuation of injectable contraceptives.	Timely reminders of fertility intentions.
***Bottlenecks Contributing to Active Discontinuation***	
4. Perception of Limited Choice Set for Continued Use: Women experience and wish to eliminate side effects from injectable contraceptives, yet they do not feel that they have viable alternatives, nor do they consider switching methods.	Expand the choice set of methods women consider to be viable options; increase and maximize HEW and WDA touch-points with women.
***Bottlenecks Contributing to Provider Behavior***	
5. Bandwidth Tax: HEWs have limited time and attention to devote to client tracking and case management. Thus, the date of clients’ follow-up appointment is not salient to HEWs at the appropriate time to provide prospective appointment reminders and retrospective follow-up for missed appointments.	Simplify case management systems to reduce the cognitive load of HEWs.

**Figure 2.**
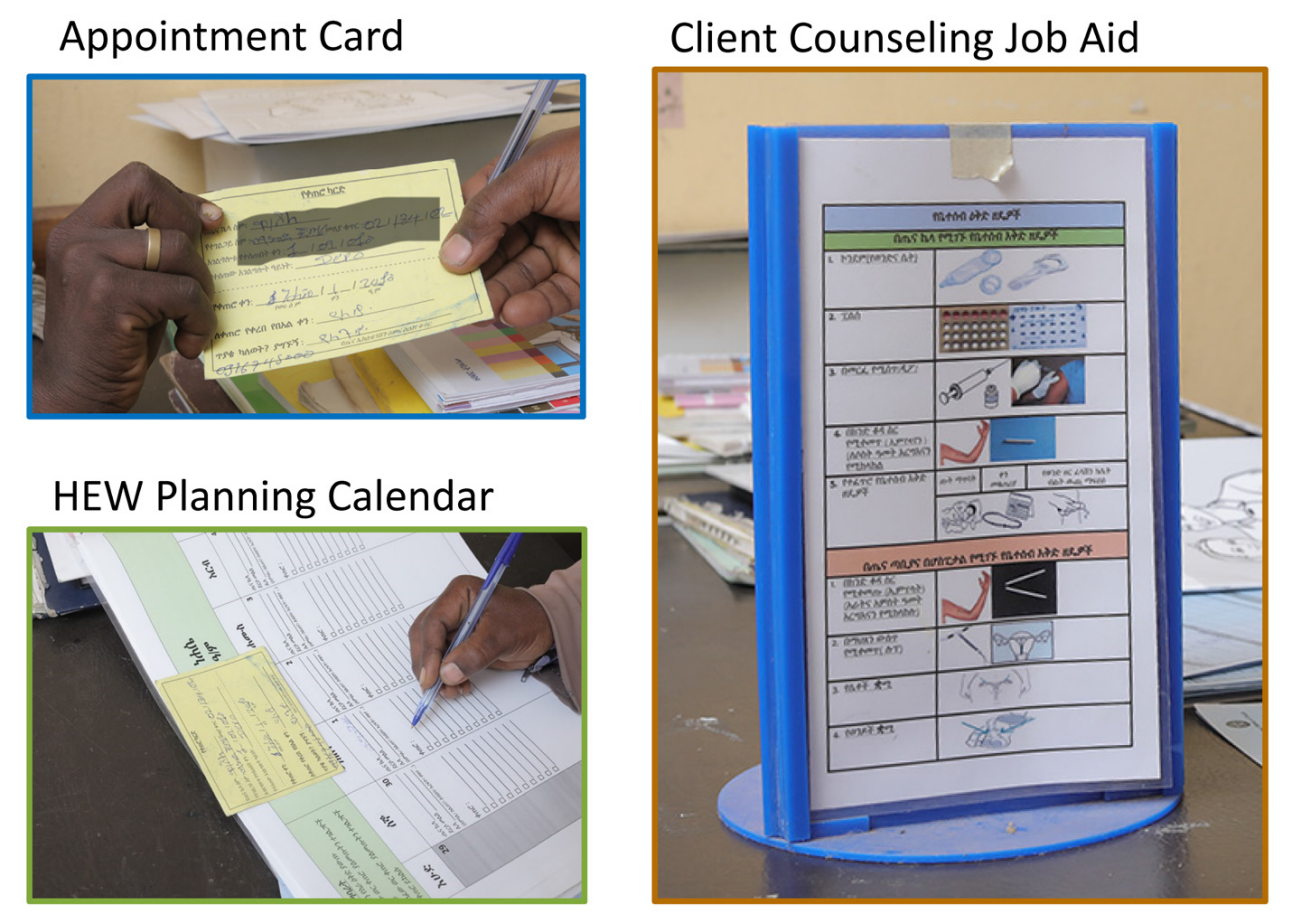
Clockwise from left: appointment card, client counseling job aid, and Health Extension Worker (HEW) planning calendar.

***Intervention design (Step 3).*** Building directly on the behavioral diagnosis and related design implications, a package of scalable interventions was selected and refined to ensure that the interventions were feasible, culturally acceptable, aligned with HEP policies, contextually appropriate, and human-centered. To ensure human-centered design, the tools were co-created with the HEWs and clients. The intervention design package included three components—a HEW planning tool located at the health post; a client counseling job aid used by HEWs during service provision; and appointment cards distributed by HEWs during service provision and used by clients ( Figure 2).

*HEW planning calendar:* The planning calendar is a desktop-based calendar, located at the health post and utilized by the HEW. The planning calendar serves as an aid for the HEW when scheduling future appointments and outreach, and it guides her toward better time management and planning.

*Client counseling job aid:* The counseling job aid is a simple guide to frame the HEW service provision interaction with clients to (1) to prompt HEWs to provide high-quality services at every interaction and to create an atmosphere in which the client feels comfortable and supported, (2) prompt the key components of family planning counseling, and (3) reduce the extent to which a provider needs to remember exact protocols for setting return appointments and managing records. Three job aids—one for counseling on available family planning methods for informed choice; one for counseling new family planning clients; and one for counseling returning clients—are displayed on three sides of a triangular tower and kept on the HEW’s counseling table.

*Appointment cards:* HEWs distribute appointment cards to all clients requiring return appointments. They remind clients about future appointments and provide critical information on what to do if they cannot make their appointments or experience health concerns in the interim.

***Test (Step 4).*** The final step of the behavioral design methodology was to test the package of interventions to assess implementation feasibility and impact on the outcomes of interest.

### Trial design

A stratified-pair cluster-randomized field trial was implemented to test the package of BE interventions. The stratification variable was the administrative region. One of the two FP intervention PHCUs (i.e., districts) from each region was randomly selected for the intervention arm, while the other served as the control arm. All 19 satellite health posts in the four intervention PHCUs received the BE intervention, while the 21 health posts in the four PHCUs of the control arm did not receive it ( Table 2). In Adinebried, Alefa, and Ebot Tirora PHCUs, the BE intervention package was initiated in February 2016; and in Sentema began in March 2016. Masking the intervention package was not possible.

**Table 2.** List of primary health care units (PHCUs) by study arm. SNNP - Southern Nations, Nationalities and Peoples’.

Study Arm	Region	District	PHCU	# of Health Posts
*Control*	Amhara	Ensaro	Lemi	3
	Oromia	Chora	Kumbabe	8
	SNNP	Yem	Fofa	7
	Tigray	Samre Sehart	Finarwa	3
*Intervention*	Amhara	Burie Zuria	Alefa	5
	Oromia	Seka-Chekorssa	Sentema	4
	SNNP	Dalocha	Ebot Tirora	5
	Tigray	Laelay Adiabo	Adinebried	5

### Trial implementation

A two-day training was developed, covering the purpose of the study and the implementation of the intervention package. All HEWs including their supervisors at the District Health Office and health centers were trained. A family planning client register was introduced at the health posts so that the study participants could be traced and interviewed to estimate the injectable contraceptive discontinuation rate. The HEWs including their supervisors in the control districts were trained to use the family planning client register.

L10K staff conducted routine supportive supervision of the study area mainly to support its broader family planning intervention.

### Study participants

The study participants were women who visited the study area health posts during the intervention period and fulfilled any of the following three criteria: (1) initiating use of injectable contraceptives; (2) returning to the use of injectable contraceptives after at least a six-month lapse; or (3) switching to injectable contraceptives from another family planning method. Those who did not meet any of these three criteria were excluded from the study.

### Outcome measurements

The primary outcome of interest was the injectable contraceptive discontinuation rate within 12 months of uptake. To measure it, first the duration of injectable contraceptive use was measured from the three survey items: (1) the month and year the eligible participant visited the health post between intervention onset and November 2016 (uptake month); (2) whether the participant was still using the injectable contraceptive without interruption at the time of the survey, or with a maximum of a month (30 days) of interruption (survey month); and (3) if the participant was not currently using the injectable contraception, then the month and year the participant had last obtained injectable contraceptive without interruption or with a maximum of one month of interruption (dropout month). The duration of injectable contraceptive use from uptake is the difference between uptake month and survey month, or uptake month and dropout month. The duration was measured in months. In a few of the dropout cases, where the participant did not accurately describe the last time she obtained an injectable contraceptive (without interruption), then the duration of use was obtained by asking how long she had used injectable contraception without interruption.

The secondary outcomes considered were mean number of correct side effects of injectable contraceptive spontaneously recalled by the participant; mean number of different types of contraceptive methods spontaneously recalled by the provider; mean number of correct actions to be taken spontaneously recalled by the participant if she experienced side effects from injectable contraceptives; whether the participant was told about the side effects of injectable contraceptives by the provider; whether the participant was told what to do if she experienced side effects; whether the participant was informed about other methods of contraception; whether the participant was told, during her last visit to the health post for injectables, about a local holiday or event as a reminder of the next appointment date; whether the participant was given an appointment card during the last visit to obtain an injectable contraceptive; and whether the participant missed an appointment for injectable contraceptives.

### Sample size

The sample size for the study depended on the caseload of the health posts and the duration of the recruitment period. Initially, it was decided that the recruitment period for the study would be six months. Based on the caseload records of the health post, it was estimated that in six months about 660 eligible participants (330 from each study arm) could be recruited. If the expected discontinuation rate of injectable contraceptives within 12 months of uptake without the BE intervention was at 34% (the level reported in the Ethiopian Demographic and Health Survey conducted in 2011) ( Central Statistical Agency and ICF, 2012), the sample size that would be obtained in six months would have 80% power to detect at least a 14 percentage-point reduction in injectable discontinuation rate with a 95% confidence level, if the cluster-randomization study design effect was 2.0 and the nonresponse rate was 5%.

### Data collection

Data collection was planned to take place 12 months after September 2016, i.e., in October 2017. However, due to competing priorities, the data collection was conducted in December 2017. Eligible participants were drawn from women of reproductive age who visited the study area health posts for injectable contraceptive services between the onset of the intervention and November 2016.

Ethical clearance for the study was obtained from each of the four Regional Health Bureaus and from the JSI Ethical Clearance Committee. Two types of questionnaires were pretested and used—one for the health post in the study area and the other for the study participants (see extended data ( Karim, 2019)). Data collection from the health posts was done in October 2017 to assess the status of the BE interventions and to assess the status of the overall health system functions using a structured questionnaire. One day of training for data collection from the health posts was provided to four public health professionals and who had previously conducted health facility surveys for L10K. The supervisors of the health post data collection were L10K regional office staff. Survey responses were electronically captured using SurveyCTO, a web-based survey data-collection platform using mobile technology ( Dobility Inc., 2018).

The study participant questionnaire was pre-coded and translated into three major local languages: Amharic, Oromia, and Tigrigna. In SNNP, where there are several local languages, the interviewers translated questions from Amharic informally. In total, 28 public health professionals were given a two-day training on collecting data from the study participants, which included practical exercises. The data collectors had previously conducted household surveys for L10K. Eight of the data collectors who had survey experience from conducting previous L10K surveys were oriented to supervise the data collection. The data collectors did not know which was the intervention arm and which was the control arm. The list of women of reproductive age who visited the study area health posts for injectable contraceptive services between the onset of the BE intervention and November 2016 was obtained from the family planning client registers. All the women in the register were visited at their households, and those who met the eligibility criteria and consented to participate were interviewed. The data collection from the study participants was done in December 2017. Survey responses were electronically captured using SurveyCTO.

***Independent variables.*** The major independent variable of interest is an indicator variable for the intervention area (study arm). The effect of the intervention was controlled for background characteristics of the participants and the health system functioning status indicators of the health posts. The background characteristics considered were age, education, number of children, age of the last child, and wealth quintile. A wealth index score was constructed for each participant’s household using principal component analysis of household possessions (electricity, watch, radio, television, mobile phone, telephone, refrigerator, table, chair, bed, electric stove, and kerosene lamp), and household characteristics (roof material, wall material, type of latrine, and source of drinking water). Households were ranked according to the wealth score and then divided into five quintiles ( Filmer & Pritchett, 2001).

The health system functioning status indicators measured for the study area health posts were population to HEW ratio of the kebele; household to active WDA team leader ratio of the kebele; number of Level 3 and Level 4 HEWs at the health post; whether the health post was closed for a month or more during the observation period; availability of contraceptives at the health post during the day of the visit; frequency of supportive supervision visits received by the health post; whether the health post maintained and updated the health management information system records; whether the health post maintained a tickler file system for family planning clients; and whether the health post had updated records for activities related to the Community-Based Data for Decision Making (CBDDM) strategy of the L10K project. Adherence to the BE intervention was measured by observing the health post for the presence of appointment cards, placement of counseling job aid on the counseling table, and use of HEWs’ planning calendar.

### Statistical analysis

All statistical analysis was done using Stata version 15.1 ( StataCorp, 2017). The availability and use of BE tools in the intervention area health posts were assessed using descriptive statistics. Then, the distributions of study participants’ background characteristics were analyzed, and statistically significant differences in the background characteristics between the study arms were assessed using Pearson’s chi-squared statistics, adjusted for clustering of the respondents within the health posts.

Distributions of the study participants according to health post characteristics were compared between study arms, and the statistically significant differences were assessed, also using adjusted Pearson’s chi-squared statistics. The characteristics of the health posts were the health system functioning status indicators described in the measurement section above.

The intervention effects on the outcomes of interest were based on intention to treat analysis. Logit regression was used when the secondary outcome variable was binary, and ordinary least squares regression when the secondary outcome variable was continuous. The analysis accounted for the stratified PHCUs, and all the independent variables described in the measurement section above. Stepwise backward selection was implemented to identify regressors for each of the outcome models with p-values set at 0.2 for variables to enter or exit the model. The study arm indicator and the indicators for the stratified PHCUs, i.e., administrative region indicators, were forced to remain in the model. Since there were only two randomization units per region, and one of them was randomly selected for the intervention, the combination of study arm and administrative region indicator variables accounted for unobserved PHCU-level variance (see PHCURandomEffects.log of the extended data ( Karim, 2019)). For each outcome, a health post-level random effects regression model was estimated using the independent variables from the backward selection process. If the health post level random effect was statistically significant (p < 0.05), then the health post-level random-effects model was the final model, and if not, then the model identified by the backward selection with robust standard errors was the final one. The goodness-of-fit of the final model was assessed using a global F-test, global Wald’s statistic, or global likelihood ratio test, depending on the estimation method for the final model. The final model was used to obtain adjusted point estimates of the mean outcome and its 95% confidence intervals by study arm. The adjusted differences in the outcomes between the two study arms and its 95% confidence intervals were also estimated.

The intervention’s effect on the primary outcome was estimated with a discrete hazard model using logistic regression ( Allison, 1982; Canette, 2016). Data were organized and analyzed using the methodology described by Canette (2016). The discrete units were months. The identification of the final model was done using the same procedure that was used to identify the models for the secondary outcomes described above. The final model was used to estimate the injectable discontinuation rate during the 12 months following uptake.

## Results

In general, the catchment populations of the health posts in the intervention areas were near twice those of the control areas. The average catchment population of the health posts in the intervention area was 7,868; for the control area it was 4,064.

Assessment of the implementation status of BE tools at the health posts indicates that 12 of the 19 health posts in the intervention area had appointment cards available on the day of the visit, while only two health posts were stocked out of the appointment cards for more than a year ( Table 3). Client counseling towers were available in all the intervention area health posts, and they were on top of each HEW’s counseling table. However, the HEWs from one of the intervention area health posts indicated that they did not use the counseling job aid. Use of HEW the planning calendar was poor. Only four of the intervention area health posts were using the planning calendar appropriately, while the rest were not using it during the four weeks preceding the survey, or they did not have the calendar for the most current year.

**Table 3.** Adherence to behavioral economics (BE) tools at the intervention area health posts during the day of the visit. HEW – Health extension workers.

Behavioral economics tools	%	# of health posts
Appointment card		
Have card	63%	12
No card since > 3 months	16%	3
No card since > 6 months	11%	2
No card since > 1 year	11%	2
Client counseling job aid		
Always	74%	14
Usually	21%	4
Never	5%	1
HEW planning calendar		
Current calendar present and updated	21%	4
Current calendar present but not updated	32%	6
Current calendar not available	47%	9

Health post records indicated that 2,490 women in the intervention area and 1,684 women in the control area visited the study area health posts between February 2016 and November 2016 to obtain injectable contraceptives ( Table 4). Among them, 335 women (13%) from the intervention area and 408 women (24%) from the control area were eligible for the study and were interviewed after obtaining consent; 688 women (28%) in the intervention area and 550 women (33%) in the control area were not eligible; 1,047 women (42%) in the intervention area and 559 women (33%) in the control area could not be located due to erroneous records; while 420 women (17%) in the intervention area and 167 women (10%) in the control area could not be reached for interview. If the eligibility rate for participating in the study among those who could not be located for an interview was similar to those who were located, then the loss to follow-up of eligible study participants was 22% in the intervention area and 18% in the control area. There were no non-responses among those who were eligible.

**Table 4.** Frequency distribution of family planning clients who visited the health posts in the study area between February 2016 and November 2016.

Category	Control # (%)	Intervention # (%)
Number eligible and interviewed	408 (24)	335 (13)
Number not eligible	550 (33)	688 (28)
Number could not be located (erroneous records)	559 (33)	1,047 (42)
Number could not be reached	167 (10)	420 (17)
Total	1,684 (100)	2,490 (100)

Table 5 compares the background characteristics of the study participants between the intervention and control areas. Compared to those of the control area, the intervention area participants were less likely to have higher education (p < 0.05); and the age of the last child was less likely to be more than three years (p < 0.001). Otherwise, participant characteristics between treatment and control areas did not differ with statistical significance.

**Table 5.** Distribution of study participants by background characteristic (%). SNNP - Southern Nations, Nationalities and Peoples’.

Sample characteristics	Control ( *N* = 408)	Intervention ( *N* = 335)	*p*-value
Age group			0.441
16–22	26	26	
22–29	41	36	
30–39	26	33	
40–49	7	6	
Education			0.043
None	56	66	
Primary	19	19	
Higher	25	16	
Age of last child			< .001
> 3 years	92	81	
≤ 3 years	7	5	
No children	2	14	
Religion *			0.480
Orthodox	60	69	
Protestant	3	0	
Muslim	38	31	
Wealth quintile			0.273
Lowest	17	24	
Second	13	29	
Middle	23	16	
Fourth	25	14	
Highest	22	17	
Region			0.393
Amhara	16	25	
Oromia	41	21	
SNNP	23	12	
Tigray	20	42	

*For the statistical test, Protestants were collapsed with Orthodox Christians

Table 6 shows the distribution of the study participants according to the health system functional status of the health posts to which they belonged. Although it appeared that compared to participants from the control areas, participants in intervention areas were more likely to be in the catchment area of health posts with better-functioning health systems (i.e., more Level 4 HEWs, higher density of active WDA team leaders, fewer contraceptive stockouts, more frequent supportive supervisory visits, more frequently updated family folders, more updated CBDDM registers, and less likelihood of closure for more than a month during the past two years), the differences were not statistically significant. However, the HEWs in the intervention area were serving a larger population. Of the participants in the intervention area, 81% were in the catchment area of health posts that had more than 2,500 people for every HEW deployed, compared to 37% in the control area (p < 0.01).

**Table 6.** Distribution of study participants according to the health system functional status indicators of the health posts. HEW – Health Extension Workers, WDA – Women’s Development Army, CBDDM – Community-Based Data for Decision making.

Indicator	Control		Intervention		*p*-value
	% of Participants ( *N* = 408)	# of Health Posts ( *N* = 21)	% of Participants ( *N* = 335)	# of Health Posts ( *N* = 19)	
Population per HEW in the kebele					0.006
≤ 2,500	63	14	9	2	
2,501 to 5,000	30	5	84	14	
> 5,000	7	1	7	3	
Number of Level 3 HEWs					0.972
0	22	5	19	3	
1	29	6	29	6	
2+	49	10	52	10	
Number of Level 4 HEW					0.564
0	53	12	34	8	
1	23	4	38	7	
2	25	5	27	4	
Households per active WDA team leader					0.204
< 50	12	3	41	7	
50-100	54	10	34	6	
> 100	34	8	25	6	
Number of methods stocked out on day of visit					0.647
0	49	9	65	11	
1	37	9	26	6	
2+	14	3	9	2	
Supportive supervisory visit received					0.164
Last month	40	7	60	10	
In last 3 months	26	6	32	6	
> 3 months	35	8	8	3	
Have family folder					
No	0	0	0	0	
Yes	100	21	100	19	
Household demographics of family folders updated in last 12 months					0.412
No	33	7	20	5	
Yes	67	14	80	14	
Family planning tickler file					0.761
Currently used	91	18	87	17	
Not currently used	3	1	8	1	
Not available	6	2	5	1	
CBDDM register updated					0.090
All updated	24	6	62	10	
Partially updated	17	4	10	2	
Not available/not updated	59	11	28	7	
Health post closed					0.310
No	93	19	98	18	
For > 1 month	7	2	2	1	

The frequency distribution of person-months of follow-up according to the independent variables can be found in Table A1 of the data repository (see extended data ( Karim, 2019)).

**Figure 3.**
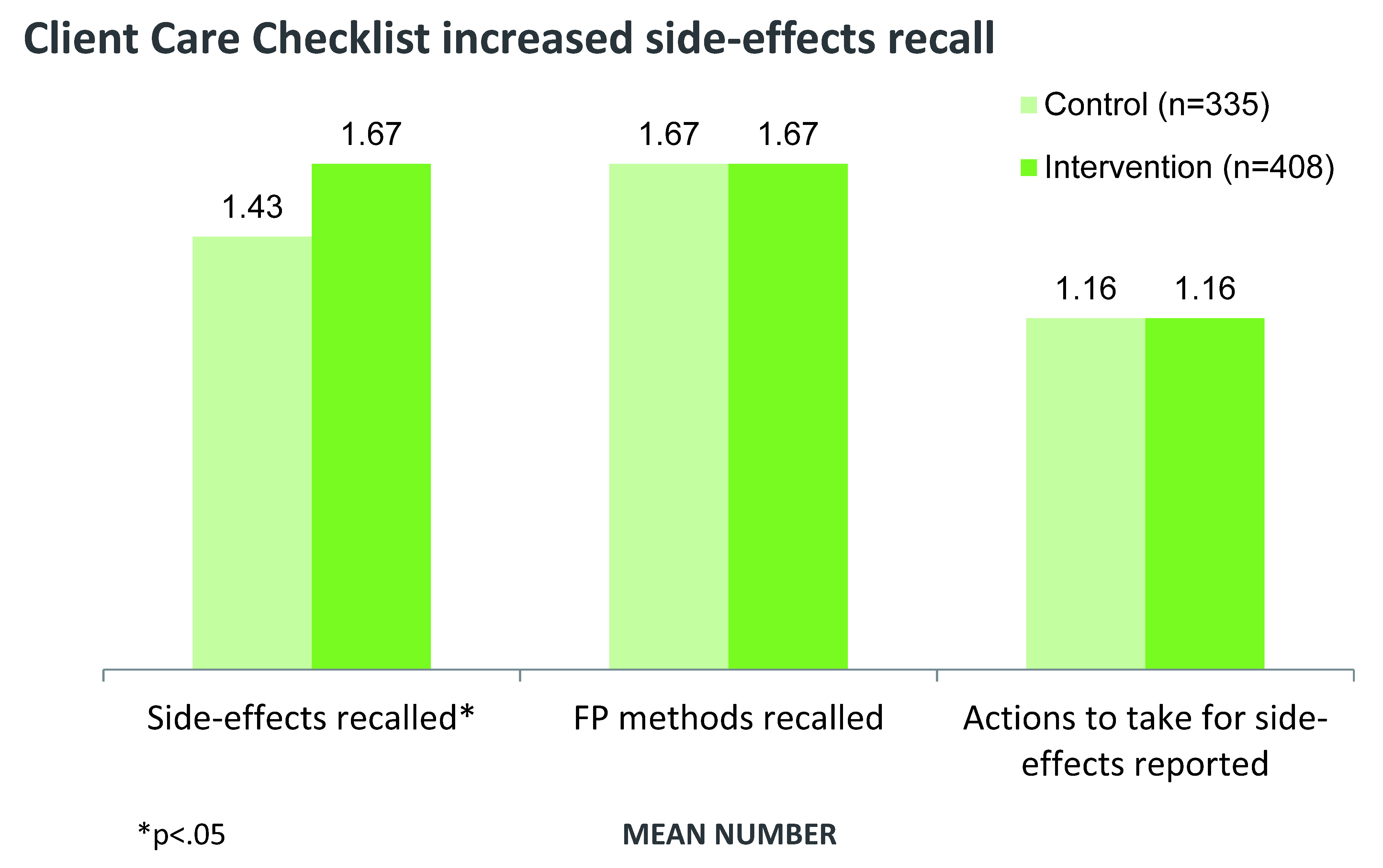
Mean number of side effects, family planning (FP) methods, and action to take for side-effects recalled by the participants.

**Figure 4.**
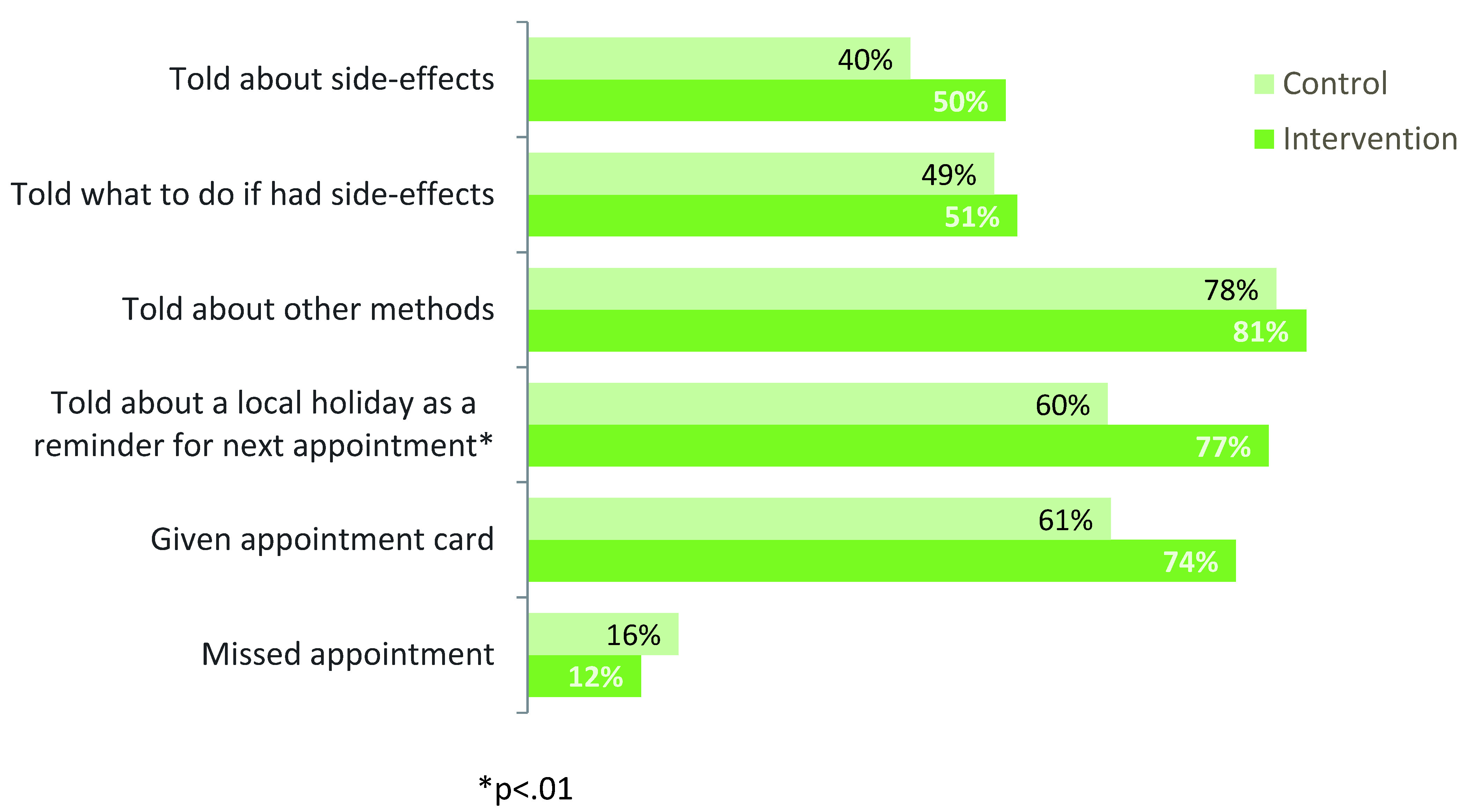
Percentage of participants who receiving method information from the provider, given appointment card and missed an appointment.

**Figure 5.**
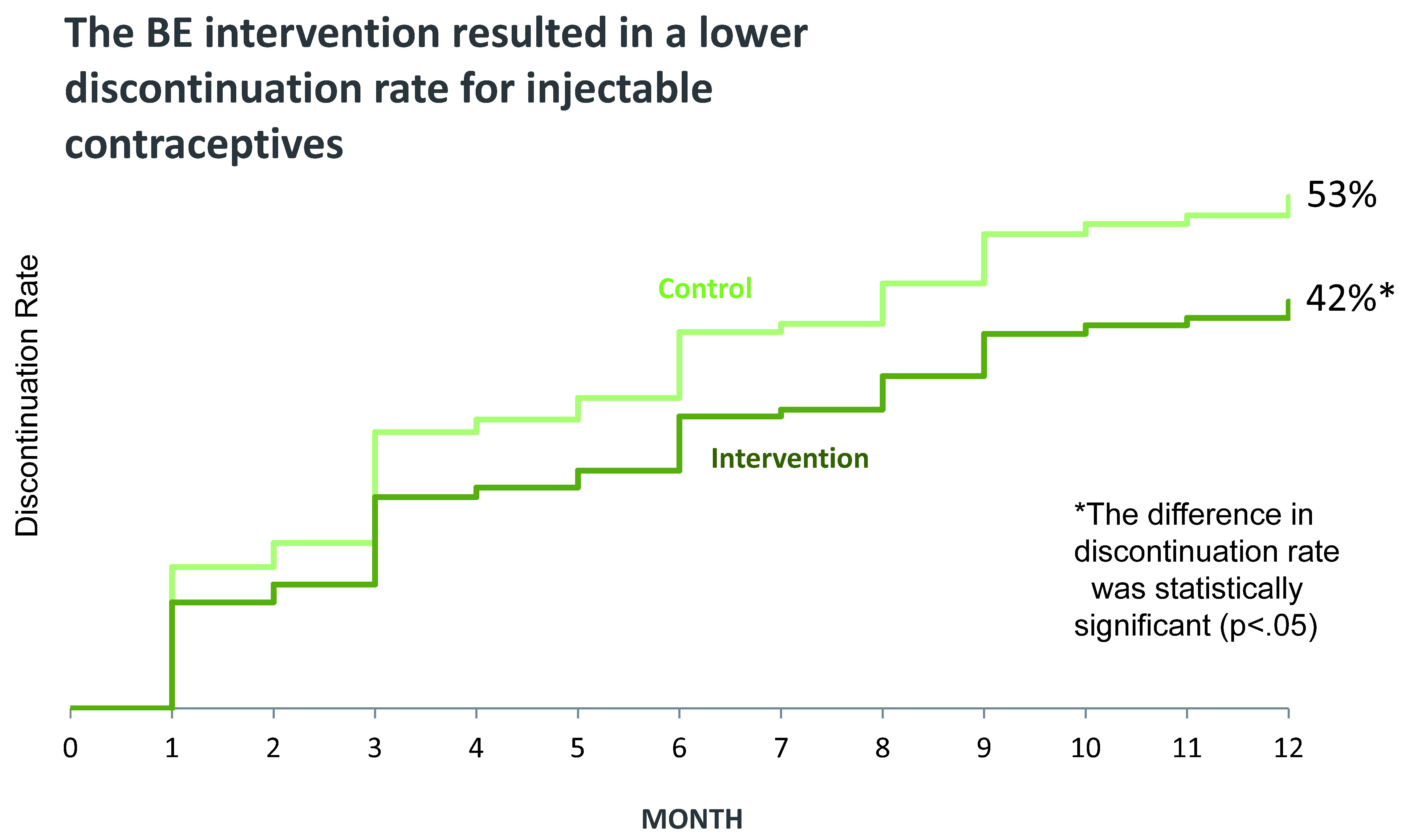
Injectable contraceptive discontinuation rate.

Table 7 gives the adjusted point estimates of the outcomes of interest by study arm and the intervention effects with 95% confidence intervals. The adjusted estimates considered the differences between the study arms observed in Table 5 and Table 6. Figure 3, Figure 4 and Figure 5 display the analysis in Table 7 using figures. The mean number of side effects from injectable contraceptive methods recalled was 0.24 higher (p < 0.05) among the intervention area participants than among those in the control areas. The proportion who recalled being told of a local holiday or event as a reminder for the next appointment for injectable contraceptives was 16.7 % points higher (95% Confidence Interval [CI]: 7.8 %, 25.6%) among the intervention area participants than among those from the control areas (p < 0.001). Although the intervention’s effects on the proportion told about side effects, proportion told what to do if they had side effects, the proportion told about other methods, the proportion given appointment card, and the proportion who missed any appointment were in the expected directions, they were not statistically significant (p > 0.1).

**Table 7.** Adjusted effects of the intervention on secondary and primary outcomes.

Outcome	Control	Intervention	Difference (95% CI)	*p*-value
Mean # of side effects of injectable contraceptive recalled	1.43	1.67	0.24 (0.05, 0.43)	0.015
Mean # of contraceptive methods available recalled	1.67	1.67	-0.00 (-0.13, 0.13)	0.961
Mean # of actions that can be taken for side effects recalled	1.16	1.16	0.00 (-0.08, 0.08)	0.999
% told about side effects	39.8	49.6	9.8 (-3.7, 23.4)	0.156
% told what to do if they had side effects	48.0	51.5	3.4 (-4.8, 11.6)	0.415
% told about other methods	78.2	80.5	2.3 (-3.8, 8.4)	0.460
% told about a local holiday or event as a reminder for appointment	60.3	77.0	16.7 (7.8, 25.6)	< 0.001
% given an appointment card	60.6	73.6	12.9 (-3.1, 28.9)	0.113
% missed any appointment for an injectable contraceptive	15.7	11.8	-3.9 (-9.2, 1.4)	0.150
% discontinued injectable contraceptive within 12 months of uptake	52.6	41.8	-10.8 (-19.3, -2.2)	0.013

The primary outcome of interest, i.e., injectable contraceptive discontinuation rate within 12 months of uptake, was 10.8 % points lower (95% CI: 2.4 %, 19.1%) among intervention area participants than among control area participants (p < 0.05) ( Table 7 and Figure 5).

Table A2 in the data repository (see extended data ( Karim, 2019)) gives the list of independent variables that were used in the final models to obtain the adjusted intervention effect estimates.

## Discussion

This is a unique study that applies a behavioral design methodology to reduce injectable contraceptive discontinuation in rural Ethiopia. The results of this study indicate that behavioral design methods are a promising approach for creating effective public health solutions. The four-stage methodology was feasibly implemented in a participatory manner alongside uninterrupted program and service delivery. We show that two of the three elements of the behavioral intervention were implemented with fidelity and that, as a package, were effective in decreasing injectable contraceptive discontinuation in the study environment, thus supporting the hypotheses generated during the behavioral diagnosis phase.

Several factors related to the internal and external validity warrant discussion. First, erroneous identification records of injectable users in the study area health posts were substantial. It is common knowledge among HEP workers, though not well documented, that some contraceptive users choose to use injectables discreetly and provide false identities to obtain services. The data collectors confirmed this assumption with the HEWs in the study area. Poor record-keeping at the health posts may also be partly responsible for the errors. Second, differential loss to follow up by study arm could have biased the study result. However, if the injectable contraceptive discontinuation rate among women who were lost to follow-up was similar to that of women who were not lost to follow-up within the respective study arms, then the estimate of the intervention effect was unbiased. Although we cannot directly test differential loss, we show that age, religion, and region of residence are all broadly similar across intervention groups. If these characteristics are the primary drivers of whether clients provide false identities, we would not expect the desire to use injectables discreetly to differ by treatment arm either. Third, the threat to the validity of the randomization is that there was no baseline on a wide range of characteristics and that some of the treatment effects are highly sensitive to the specific controls used. To validate these treatment effects (especially for the primary outcome) we rely on the backward selection process using variables in Table 5 and Table 6, and the model specification derived from it, as most appropriate and that unobserved differences do not drive the measured treatment. Fourth, the design of participant recruitment and eligibility may have introduced sample selection bias. Specifically, participants were eligible for the study if they were new injectable users (defined as someone new to family planning, switching from one method to another, or who had taken a hiatus from family planning for six months or more). Because the intervention reduced discontinuation and encouraged dissatisfied users to switch methods, it is possible that the study participants who were included during the latter part of the enrollment period were different between the study arms. The intervention arm may have included more women who were switching to injectable contraceptives from a prior method. Table 5 and Table 6 show that for both individual- and provider-level characteristics, there is broad balance between treatment and control participant. However, we are unable to completely eliminate the possibility of this bias. Fifth, having more than 12 months of the observation period, there was an opportunity for health system factors (for example, injectable supply and availability of HEWs) to mask the effects.

Injectable discontinuation rates in both arms of the study were much higher than initially expected. The difference in the representativeness and measurement method of the discontinuation rate between the expected (nationally representative data with a five-year recall period) and the observed (which was in eight districts with one-year recall period) may partly explain it. It could be that the respective Regional Health Bureaus purposely selected low performing districts for L10K to support the family planning program. Nonetheless, variations in the injectable discontinuation rate between geography is observed in Ethiopia. An independent study in 2014 in Agarfa district, Oromia, report significant higher rates of injectable discontinuation (67%) than the national average ( Bekele *et al*., 2015).

Results on the primary outcome of this study show that the overall intervention package was successful in reducing injectable contraceptive discontinuation. Examining the secondary outcomes of interest sheds light on the distinct components of the intervention package and the accuracy of the behavioral diagnosis insights. Study results indicate that the appointment cards and the client counseling job aid were utilized in the intervention areas, whereas the planning calendar was generally not utilized by HEWs. Before the study, the existing family planning client tracking system for HEWs was the tickler filing system to track defaulters ( Chewicha & Azim, 2013). The behavioral diagnosis revealed that it was not a useful tool to help HEWs provide prospective appointment reminders for the next dose of injectable contraceptives nor was it an effective tool to track missed doses. The planning calendar was designed to help the HEWs provide prospective follow-up reminders to the FP clients. However, it is unlikely that the HEW planning calendar had any effect on the primary outcome, mainly because nine of 19 intervention area health posts did not have the most recent planning calendar, and among the ten health posts that had copies of the most recent planning calendar, six were not using them. One of the possible reasons for not using the planning calendar could be that the HEWs were already overwhelmed by the existing recording requirements of the HEP, and the planning calendar was an extra recording burden for them. The planning calendar was a parallel system to the HEWs’ existing record-keeping efforts.

Study participants in both arms reported receiving appointment cards from the HEWs during the last time they visited the health post to obtain injectable contraceptives. This was not surprising because, during the problem definition and behavioral diagnosis exercises, it was noticed that some HEWs provided injectable contraceptive clients a paper note as a reminder for the next appointment. Thus, formal appointment cards institutionalized an already used ad-hoc strategy used by the HEWs as one of the intervention tools. Additionally, while providing the appointment card and counseling clients, the HEWs in the intervention area were advised to relate the appointment date for the next injectable dose to a local holiday or an event and to write it down in the appointment card. Thus, as expected, it was found that the intervention area study participants were more likely to recall being told about a local holiday as a reminder for the appointment date. Given it was an already-used practice by HEWs in this context, associating the return appointment date with a local event or holiday likely increased appointment salience and recall, even without the availability of printed appointment cards. The use of the formalized appointment card validates the participatory and user-centered behavioral design approach in which solution concepts are sourced from the end-users themselves and designs build off of existing practices, habits, and patterns of behavior.

Recall of the side effects of injectable contraceptives was also higher among the intervention area participants, which is consistent with HEWs using the client counseling job aid component of the behavioral package. Although L10K provided refresher training to HEWs in both study arms on the family planning client care job aid provided by the national program, this job aid was long and cumbersome for counseling clients, and rarely used by HEWs. The behavioral design process made the original job aid more concise by increasing the salience of key messages and reducing the amount of information included. The new job aid was mounted on a desktop tower (rather than presented in a book or table tent that required page-turning) to be readily accessible and salient during client counseling. These two design adaptations may have made the job aid more usable and effective for HEWs.

It is interesting to note that using the appointment card and the client counseling job aid were within the routine workflow of the HEWs, but the planning calendar required an alteration of the HEWs’ workflow. Thus, HEWs complied with the use of appointment cards and client counseling job aid, but not with the use of the planning calendar. This is a critical insight for solution design—both within global health programs and across behavioral design more generally. Designs that introduce new tools or processes within an existing workflow, require an additional step or action, or require the shifting of habits, should be carefully considered and heavily pre-tested prior to implementation.

The study design cannot distinguish whether the appointment card or the counseling job aid was more effective. Ideally, the tools could have been tested one by one, with a shorter duration of observation, to identify the effectiveness of each. The one-year observation period required to measure the primary outcome for the study prevented the use of rapid tests one by one. This could be a blessing because it is possible that the appointment card and counseling job aid had a synergistic effect. Nonetheless, a shorter testing duration would have been beneficial to keep policy-makers interested, and to convert research findings into policy more rapidly.

The behavioral diagnosis pointed to both provider-side and client-side behavioral bottlenecks contributing to discontinuation of injectable contraceptives. Given the available data from this study, it is difficult to disentangle exactly how the intervention package interacted with each specific bottleneck. However, we can offer some observations. On the provider-side, the diagnosis revealed that HEWs experienced a condition of scarcity driven by heavy workloads, time-intensive tasks, and a high number of services to provide. This context of limited time and attention may have led HEWs to tunnel on more pressing issues, putting aside lower priority client tracking and case management. Low utilization of the planning calendar (which would have required an additional step or process by the HEWs) reinforces this insight. Though the planning calendar was designed to ease HEWs bandwidth constraints, it does not seem to have been effective in this regard. The client counseling job aid and the appointment card, while also targeting client-side bottlenecks, also served to address the HEWs taxed bandwidth. These tools acted as salient reminders and physical cues prompting providers to employ a specific protocol and provide key pieces of information during appointments.

On the client-side, the diagnosis highlighted behavioral bottlenecks contributing to both passive and active discontinuation. The appointment card targeted passive discontinuation by serving as a physical reminder for a future appointment and providing a salient memory cue (the closest holiday to the appointment) to help clients remember the appointment date. It may have also targeted active discontinuation by including personalized information from the HEW (name and phone number), thereby offering a solution if the client was actively considering discontinuing. The client counseling job aid targeted active discontinuation by ensuring that the HEW provide key messages around side effects, continued use of the method, and what to do if the client was dissatisfied with the method or missed a follow-up appointment. Further exploration would be necessary to understand in greater detail exactly how the intervention package targeted the five individual bottlenecks identified in the diagnosis.

Across contexts, dissatisfaction is the dominant reason for stopping a modern, reversible method ( World Health Organization, 2012). Dissatisfaction may stem from side effects, among other reasons ( Bradley *et al*., 2009). The client counseling job aid prompted HEWs to discuss with clients both side effects and what to do if side effects were experienced. The results show that clients in the intervention area were able to recall a higher number of side effects than the control area. However, across the intervention and control areas, there was no significant difference in client recall of the actions that could be taken for side effects. Given the reduction in discontinuation in the intervention area as a result of the intervention package, this represents an interesting data pattern. Perhaps the awareness of potential side effects alone was a meaningful factor in reducing discontinuation. Behavioral research shows that not only do we react more strongly to negative events than positive ones ( Baumeister *et al*., 2001; Taylor, 1991), our responses are heightened in response to surprising or unexpected events as compared to expected events ( Shepperd & Mcnulty, 2002). The suggestion that the mere awareness of potential side effects may lessen the intensity of a negative experience is an area for future practical exploration.

## Conclusion

The overall findings of this study suggest two primary conclusions: first, that the behavioral design methodology is a useful approach for solving persistent behavioral challenges in family planning programs, and second, that two components of the intervention package (the appointment card and the client counseling job aid) are promising solutions for enhancing family planning programs in this low-resource, rural setting in Ethiopia. The behavioral design methodology was successfully implemented by a multidisciplinary team to identify novel behavioral insights around injectable discontinuation, design a context-appropriate solution, and test the effectiveness of the intervention package. Two of the three behavioral tools in the intervention package were consistently utilized by HEWs in the intervention area. A reduction in injectable discontinuation in the intervention area of over 10 percentage points can be attributed to the design package. In addition, increases in secondary outcomes such as the number of side effects recalled and use of a local holiday or event as an appointment reminder were consistent with the behavioral intervention being utilized effectively to reduce discontinuation.

The appointment card and the client counseling job aid emerged as the two tools that HEWs most consistently used in service provision and appear to seamlessly integrate into their routine workflow. This finding is critical for behavioral design generally. Where possible, new solutions must seamlessly integrate into existing patterns of behavior, workflows, or processes. If new solutions being introduced require new habits, processes, or steps, they may need additional scaffolding and support to ensure successful utilization.

We show that the behavioral design methodology is a practical approach for identifying relevant insights and designing targeted solutions. The study’s results indicate that insights from behavioral economics can improve family planning programming and outcomes. The resulting intervention package reduced injectable discontinuation, likely reducing unintended pregnancies and improving participant health and autonomy.

## Consent

During participant recruitment, after eligible participants received services at a health facility, HEWs informed them about the study and the data collection activities. They were given the opportunity to ask any questions about the study and its participants, and then were asked if they consented to participate. HEWs were supposed to have first asked for verbal consent and then supply a written consent form to record participant contact information for the follow-up interview. All HEWs reported having administered and secured verbal consent to all eligible study participants. However, very few paper consent forms were retrieved from health posts. This implementation failure presents an unknowing violation of client privacy. Learning from this experience will inform how consent is secured in future activities.

Therefore, for the FP client follow-up survey, verbal consent was sought and documented by the interviewers. If the respondent was under 18 years old, then consent was sought from her husband, parents or guardian. As it was expected that most of the respondents could not read or write, written consent was not sought. Only when consent was given by the respondent, the electronic data collection tool allowed to continue with the survey.

Data collection from health posts was considered a structured routine supportive supervisory visit. Data was collected mainly through observations and review of health post records and extraction of administrative data. However, verbal consent from the HEW or HEWs (when more than one HEW was present during health post visit) were obtained before continuing with data collection from the health post.

The consent forms for the FP client follow-up and health posts data collection were part of the data collection tool and can be accessed with the data repository.

Ethical approval was obtained from the ethical review boards of the Regional Health Bureaus of Amhara, Oromia, SNNP and Tigray regions and from the ethical review board of JSI Research & Training Institute, Inc. The ethical approvals can be accessed with the data repository.

This study was retrospectively registered with the International Standard Randomized Controlled Trials Number registry ( ISRCTN17390653) on the 10 April 2019.

## Data availability

### Underlying data

Zenodo: Behavioral economics approach to reduce injectable discontinuation rate in rural Ethiopia. http://doi.org/10.5281/zenodo.3529744 ( Karim, 2019)

This project contains the following underlying data:

- BE health post fidility.csv (Health post survey data in comma-separated values format)
- BE_client_interview.csv (Study participants’ interview data in comma-separated values format)
- hp_pop2018.csv (Contains the population sizes of the health post catchment areas in comma-separated values format)
- master.do (Stata do- or syntax-file that implements the rest of the Stata do-files sequentially)
- hp_InterventionFidility.do (Stata do-file that reproduces the analysis presented in Table 3 and creates the independent variables of Table 6 and also used for the multi-variate analysis presented in Table 7)
- BE_client_interview1.do (This and the next Stata do-file reproduces the analysis presented in Table 5, Table 6 and Table 7)
- BE_client_interview2.do
- PHCURandomEffects.do (This Stata do-file runs the final models of the study with PHCU as a random factor)
- PHCURandomEffects.log (Output file for PHCURandomEffects.do)

### Extended data

Zenodo: Behavioral economics approach to reduce injectable discontinuation rate in rural Ethiopia. http://doi.org/10.5281/zenodo.3529744 ( Karim, 2019)

This project contains the following extended data:

- ClientInterviewQuestionnaire.pdf (Questionnaire used to interview the study participants)
- HealthPostQuestionnaire.pdf (Questionnaire used to obtain data from the health posts of the study area)
- Tables A1 and A2.docx (Table A1 presents person-months of retrospective follow-up of the study participants by the independent variables considered for the discrete hazard model that estimated injectable discontinuation rate. It also includes the name of the variables constructed for analysis. Table A2 presents the independent variables used in each of the models presented in Table 7)

### Reporting guidelines

Zenodo: CONSORT checklist and diagram for “Application of behavioral economics principles to reduce injectable contraceptive discontinuation in rural Ethiopia: A stratified-pair, cluster-randomized field trial”. http://doi.org/10.5281/zenodo.3236003 ( Karim, 2019)

Data are available under the terms of the Creative Commons Attribution 4.0 International license (CC-BY 4.0).
