# Supplementary material for: Application of behavioral economics principles to reduce injectable contraceptive discontinuation in rural Ethiopia: A stratified-pair, cluster-randomized field trial
Source: Gates Open Res. 2019 Nov 20;3:1494. Originally published 2019 Jun 13. [Version 2] doi: 10.12688/gatesopenres.12987.2 (PMC7416084; doi:10.12688/gatesopenres.12987.2)
Supplement: Peer review history

# Peer review: Reviewer response for version 2

**DOI:** 10.21956/gatesopenres.14235.r28263

**Published:** 2020-08-07

**Authors:** Margaret McConnell

**Affiliations:** 1 Department of Global Health and Population, Harvard T.H. Chan School of Public Health, Boston, MA, USA

I have reviewed the revised manuscript and I have no additional comments.

Is the work clearly and accurately presented and does it cite the current literature?

Partly

If applicable, is the statistical analysis and its interpretation appropriate?

Partly

Are all the source data underlying the results available to ensure full reproducibility?

Yes

Is the study design appropriate and is the work technically sound?

Yes

Are the conclusions drawn adequately supported by the results?

Yes

Are sufficient details of methods and analysis provided to allow replication by others?

Yes

Reviewer Expertise:

NA

I confirm that I have read this submission and believe that I have an appropriate level of expertise to confirm that it is of an acceptable scientific standard.

# Peer review: Reviewer response for version 2

**DOI:** 10.21956/gatesopenres.14235.r28264

**Published:** 2019-12-03

**Authors:** Jennifer Wesson

**Affiliations:** 1 IntraHealth International, Chapel Hill, NC, USA

Thank you for your revisions. You have addressed all of my comments.

Is the work clearly and accurately presented and does it cite the current literature?

Yes

If applicable, is the statistical analysis and its interpretation appropriate?

Yes

Are all the source data underlying the results available to ensure full reproducibility?

Yes

Is the study design appropriate and is the work technically sound?

Yes

Are the conclusions drawn adequately supported by the results?

Partly

Are sufficient details of methods and analysis provided to allow replication by others?

Yes

Reviewer Expertise:

Family planning, epidemiology

I confirm that I have read this submission and believe that I have an appropriate level of expertise to confirm that it is of an acceptable scientific standard.

# Peer review: Reviewer response for version 1

**DOI:** 10.21956/gatesopenres.14094.r27399

**Published:** 2019-07-08

**Authors:** Margaret McConnell

**Affiliations:** 1 Department of Global Health and Population, Harvard T.H. Chan School of Public Health, Boston, MA, USA

I commend the researchers on an important study and well-written manuscript. I provide a few suggestions below. I believe these should be readily addressable:

  

1. In my mind it would be useful to more thoroughly discuss other relevant literature on reminders, job aids and the building blocks of the intervention more thoroughly (particularly those that take a similar behavioral approach). This paper by McConnell *et al.* (2018 ^1^) is one example but placing the intervention within a broader context in both the introduction and discussion section would be valuable.
2. I am not clear on a few key elements of the design and analysis. First, which outcomes were registered as primary? Second, how does the analysis account for the clustering in the randomization? It would be useful to try different methods here including potentially randomization inference to obtain accurate standard errors.
3. It would be helpful to discuss potential endogeneity in the sample selection process. For example, if the interventions reduced discontinuation this would seem to suggest that the sample of individuals enrolled into the study would differ between arms as discontinuation was one of the criteria for eligibility. It would be useful to clarify and discuss this issue.
4. It appears that the rate of discontinuation was higher than expected. It would be helpful to discuss this more explicitly and to place in the context of the broader literature.

I also have some more minor points:

- In response to this sentence: "The possible bias due to loss to follow-up was minimized by the study design and by the application of regression methods" - I'm not clear on why regression methods would help with bias?
- "This was not surprising" seems too editorial for a results section - would suggest editing this to just include findings.

Is the work clearly and accurately presented and does it cite the current literature?

Partly

If applicable, is the statistical analysis and its interpretation appropriate?

Partly

Are all the source data underlying the results available to ensure full reproducibility?

Yes

Is the study design appropriate and is the work technically sound?

Yes

Are the conclusions drawn adequately supported by the results?

Yes

Are sufficient details of methods and analysis provided to allow replication by others?

Yes

Reviewer Expertise:

Global Health, behavioral economics

I confirm that I have read this submission and believe that I have an appropriate level of expertise to confirm that it is of an acceptable scientific standard, however I have significant reservations, as outlined above.

## References

[1] : Free contraception and behavioural nudges in the postpartum period: evidence from a randomised control trial in Nairobi, Kenya. *BMJ Glob Health*.2018;3(5) : 10.1136/bmjgh-2018-000888 e000888 10.1136/bmjgh-2018-000888 30364345PMC6195134

# Response

**Published:** 2019-11-12

**Authors:** Ali Karim

Point-by-point response to Margaret McConnell, ****Department of Global Health and Population, Harvard T.H. Chan School of Public Health, Boston, MA, USA

I commend the researchers on an important study and well-written manuscript. I provide a few suggestions below. I believe these should be readily addressable:

1. In my mind it would be useful to more thoroughly discuss other relevant literature on reminders, job aids and the building blocks of the intervention more thoroughly (particularly those that take a similar behavioral approach). This paper by McConnell et al. (2018) is one example but placing the intervention within a broader context in both the introduction and discussion section would be valuable.

**Response: Agreed. Updated the introduction and discussion sections to place the current study within the broader context of the behavioral science literature. In the introduction, we clarified that behavioral barriers were likely at play in Ethiopia and discussed recent relevant behavioral economics literature that informed the building blocks of the intervention. In the discussion, we identified other behavioral science research to help explain the results of the study.**

 

1. I am not clear on a few key elements of the design and analysis. First, which outcomes were registered as primary? Second, how does the analysis account for the clustering in the randomization? It would be useful to try different methods here including potentially randomization inference to obtain accurate standard errors.

**Response: The primary outcome is injectable contraceptive discontinuation rate within 12 months of uptake. To make it explicit, we made edits throughout the manuscript and now refer to the other outcomes as secondary instead of intermediate outcomes.**

**The clustering of the randomization units (i.e., PHCUs or districts) is accounted for in the models we specified. Since there were only two randomization units within each region and one of them was randomly allocated to the intervention arm, the indicator variables for administrative regions and study arm accounted for PHCU-level clustering (see PHCURandomEffects.log of the extended data (Karim 2019)). Thus, based on the conclusion that the combination of administrative region and study arm indicator variables explained the PHCU-level unobserved variance, we estimated models considering only health posts as random factors. Nonetheless, we now use robust standard errors if the health post-level random-effects model were not significant. We update the analysis section and Table 7 accordingly.**

 

1. It would be helpful to discuss potential endogeneity in the sample selection process. For example, if the interventions reduced discontinuation this would seem to suggest that the sample of individuals enrolled in the study would differ between arms as discontinuation was one of the criteria for eligibility. It would be useful to clarify and discuss this issue.

**Response: The discussion on sample selection bias is now included in the discussion section. Specifically, we added the following: “Fourth, the design of participant recruitment and eligibility may have introduced sample selection bias. Specifically, participants were eligible for the study if they were new injectable users (defined as someone new to family planning, switching from one method to another, or who had taken a hiatus from family planning for six months or more). Because the intervention reduced discontinuation and encouraged dissatisfied users to switch methods, it is possible that the study participants who were included during the latter part of the enrollment period were different between the study arms. The intervention arm may have included more women who were switching to injectable contraceptives from a prior method. Tables 5 and 6 show that for both individual- and provider-level characteristics, there is broad balance between treatment and control participant. However, we are unable to completely eliminate the possibility of this bias.”**

 

1. It appears that the rate of discontinuation was higher than expected. It would be helpful to discuss this more explicitly and to place in the context of the broader literature.

**Response: In the discussion section, we now note that the discontinuation rate observed in our study was much higher than expected. We also provide a few insights on it. Specifically, we note: “Injectable discontinuation rates in both arms of the study were much higher than initially expected. The difference in the representativeness and measurement method of the discontinuation rate between the expected (nationally representative data with a five-year recall period) and the observed (which was in eight districts with one-year recall period) may partly explain it. It could be that the respective Regional Health Bureaus purposely selected low performing districts for L10K to support the family planning program. Nonetheless, variations in the injectable discontinuation rate between geography is observed in Ethiopia. An independent study in 2014 in Agarfa district, Oromia, report significantly higher rates of injectable discontinuation (67%) than the national average (Bekele et al., 2015).”**

I also have some more minor points:

- In response to this sentence: "The possible bias due to loss to follow-up was minimized by the study design and by the application of regression methods" - I'm not clear on why regression methods would help with bias?

**Response: Agree, we do not know if any of the bias due to loss to follow-up was accounted for by the regression models. We delete the sentence.**

 

- "This was not surprising" seems too editorial for a results section - would suggest editing this to just include findings.

**Response: Agree. Updated the results section of the abstract.**

# Peer review: Reviewer response for version 1

**DOI:** 10.21956/gatesopenres.14094.r27396

**Published:** 2019-07-02

**Authors:** Jennifer Wesson

**Affiliations:** 1 IntraHealth International, Chapel Hill, NC, USA

- The article is a combination of the description of how they used behavioral economics to design an intervention and how successful the intervention was. This is my main concern/confusion about the paper. Is it a paper highlighting BE, or a paper highlighting a successful intervention package? Sometimes it seems it is one way and sometimes it is the other way. For example, the discussion section largely describes the effect of the intervention and not the implications of using BE. Whereas, the conclusion largely seems to be about using BE approaches. If the paper is going to cover both subjects, then the discussion and conclusion should, as well.
- The discussion section is weak, in my opinion. It is largely just a repeat of what the results are. The discussion section is always the hardest to write. There doesn't seem to be a lot of original thinking in this paper's discussion. What does it mean that the intervention was statistically significant? Are there more provider-behavior issues than client-behavior? How does the intervention tie back to the diagnosis?
- One question I have is whether clients agreed to be contacted by the study personnel. It sounds as if data collectors went through client registers and then hunted the clients down. This seems to be a breach of the clients' privacy. I know the study received ethical approval, but I would think about this question going forward in other research studies.
- Finally, you could delete tables 5 and 6. Their import could be said in just a couple of sentences.
- All in all, a very interesting paper. I enjoyed reading it.

Is the work clearly and accurately presented and does it cite the current literature?

Yes

If applicable, is the statistical analysis and its interpretation appropriate?

Yes

Are all the source data underlying the results available to ensure full reproducibility?

Yes

Is the study design appropriate and is the work technically sound?

Yes

Are the conclusions drawn adequately supported by the results?

Partly

Are sufficient details of methods and analysis provided to allow replication by others?

Yes

Reviewer Expertise:

Family planning, epidemiology

I confirm that I have read this submission and believe that I have an appropriate level of expertise to confirm that it is of an acceptable scientific standard, however I have significant reservations, as outlined above.

# Response

**Published:** 2019-11-12

**Authors:** Ali Karim

Point-by-point responses to Jennifer Wesson, IntraHealth International, Chapel Hill, NC, USA

- The article is a combination of the description of how they used behavioral economics to design an intervention and how successful the intervention was. This is my main concern/confusion about the paper. Is it a paper highlighting BE, or a paper highlighting a successful intervention package? Sometimes it seems it is one way and sometimes it is the other way. For example, the discussion section largely describes the effect of the intervention and not the implications of using BE. Whereas, the conclusion largely seems to be about using BE approaches. If the paper is going to cover both subjects, then the discussion and conclusion should, as well.

**Response: We have revised the abstract and updated the discussion and conclusion sections to reflect that the article is a combination of a) how we used behavioral economics to design intervention and b) how successful the intervention was.**

 

- The discussion section is weak, in my opinion. It is largely just a repeat of what the results are. The discussion section is always the hardest to write. There doesn't seem to be a lot of original thinking in this paper's discussion. What does it mean that the intervention was statistically significant? Are there more provider-behavior issues than client-behavior? How does the intervention tie back to the diagnosis?

**Response: The discussion section has been expanded to further explain the intervention results, the associated provider and client-side behaviors, the relevance of diagnosis findings, and areas for future exploration.**

 

- One question I have is whether clients agreed to be contacted by the study personnel. It sounds as if data collectors went through client registers and then hunted the clients down. This seems to be a breach of the clients' privacy. I know the study received ethical approval, but I would think about this question going forward in other research studies.

**Response: Written or verbal consent, depending on literacy, to participate in the study was sought from all family planning clients visiting the health post in both the study arms during the recruitment period (February 2016-November 2016). However, the consent records were unable to be retrieved from health posts. Thus, consent was sought again during the follow-up survey, which could have breached client privacy in a few cases. It is an important lesson for future research to take measures to maintain client consent records.**

 

- Finally, you could delete tables 5 and 6. Their import could be said in just a couple of sentences

**Response: We debated whether to keep the tables in the body of the paper or move them to the Appendix and ultimately decided to keep them in the main manuscript as it is likely that some of the paper’s audience would like to see an assessment of the balance between the two study arms to determine randomization validity.**

 

- All in all, a very interesting paper. I enjoyed reading it.

**Response: Thanks!**
